# Immunotherapy as a new perspective for the therapy of esophageal cancer

**DOI:** 10.1515/iss-2023-0023

**Published:** 2024-09-09

**Authors:** Yvonne Huber, Markus Moehler, Anica Högner

**Affiliations:** Department of Medicine I, University Medical Center of the Johannes Gutenberg University Mainz, Mainz, Germany; Klinik für Hämatologie, Onkologie und Palliativmedizin, 27695 Vivantes Klinikum im Friedrichshain, Berlin, Germany

**Keywords:** esophageal cancer, immunotherapy, SCC, EGC

## Abstract

The therapeutic landscape in nearly every therapeutic line in advanced/metastatic patients with squamous cell carcinoma (SCC) and esophagogastric adenocarcinoma (EGC) is enriched by recent approvals of immune checkpoint inhibitors (ICIs). In curative intended therapy, patients without pathological residual disease of SCC or GEJ (esophagogastric junction) cancer after preoperative chemoradiation and complete resection have access to adjuvant immunotherapy (independent of PD-L1 (programmed cell death protein 1) status, nivolumab, CHECKMATE 577). For metastatic SCC in the first-line, nivolumab combined with chemotherapy or with ipilimumab (TPS (tumor proportion score) ≥1 %, SCC, CHECKMATE 648) are approved, as well as second-line nivolumab alone regardless of PD-L1 status (ATTRACTION 03). For both, locally advanced or metastatic SCC and EGC, chemotherapy with pembrolizumab is available for patients with CPS (combined positive score) ≥10 (KEYNOTE 590) and for adenocarcinoma with nivolumab (CPS ≥5, CHECKMATE 649). Recent added approvals are chemotherapy with pembrolizumab in CPS ≥1 patients (KEYNOTE 859) and the addition of trastuzumab for personalized therapy in HER-2 positive/CPS ≥1 gastric and GEJ patients (KEYNOTE 811).

## Introduction

Esophageal cancers are often advanced and metastatic at the time of diagnosis. Innovative multimodal therapy regimens have significantly improved the prognosis of esophageal carcinomas within the last years. Immune checkpoint inhibitors play an increasing role in every line of therapy and constitute a well tolerable and promising therapy option. Immune checkpoint inhibitors can be classified in gastrointestinal tumors depending on the target protein on the tumor or immune cells into three different groups: the anti-PD-1 antibodies nivolumab, pembrolizumab, tislelizumab, camrelizumab, sintilimab, toripalimab (T cells); the anti-PD-L1 antibodies atezolizumab, avelumab, and durvalumab (cancer cells, dendritic cells); and the anti-CTLA-4 antibody ipilimumab (T cells). Especially the combination of chemo- and immunotherapy with a possible synergistic effect of T-cell recruitment and activation is the object of current research.

## Curative immunotherapy

Before immunotherapy entered the established standard therapy regimens, curative intended therapy for SCC consisted of definitive chemoradiation for esophageal cancer in the upper third and neoadjuvant chemoradiation followed by resection for esophageal cancer in the lower two thirds [1]. In EGC, perioperative chemotherapy was recommended, whereas therapy of locally advanced adenocarcinomas is currently independent of the HER2 status.

Based on the results of the Checkmate 577 study, for patients with SCC and GEJ-cancer who received neoadjuvant (radio-)chemotherapy and R0 resection with remaining residual pathologic disease (≥ypT1 or ≥ypN1), an adjuvant therapy with nivolumab is recommended and should be performed for 1 year. Placebo-controlled adjuvant application of nivolumab revealed both, a significant prolongation of the disease-free survival (DFS) (22.4 vs. 11.0 months, Hazard Ratio (HR) 0.69 [96.4 % CI 0.56–9.86]; p<0.001) and the metastatic-free survival [2].

## Palliative first-line immunotherapy

In the past, systemic chemotherapy was recommended in the palliative situation. In SCC, chemotherapy with platin and 5-fluorouracil (FU) showed a median overall survival (OS) of about 10 months [3].

With the addition of immunotherapy, the prognosis could be improved significantly: in patients with advanced SCC, the combination of nivolumab and chemotherapy (cisplatin + FU) results in significantly longer OS than chemotherapy alone (13.2 vs. 10.7 months, HR 0.74 [99.1 % CI 0.58–0.96]; p=0.002). In patients with TPS (tumor proportion score) ≥1 % the benefit was even more pronounced with a median OS of 15.4 vs. 9.1 months, HR 0.54 [99.5 % CI 0.37–0.80], p<0.0001. Further, the dual immune checkpoint blockade with nivolumab and ipilimumab compared with chemotherapy only could achieve significantly longer overall survival among patients with TPS ≥1 % (13.7 vs. 9.1 months, HR 0.64 [98.6 % CI 0.46–0.90]; p=0.001) and in the overall population (12.7 vs. 10.7 months, HR 0.78 [98.2 % CI 0.62–0.98]; p=0.01) (CHECKMATE 648) [4]. Based on these data, first-line approval was granted in the EU recently in May 2022 for both nivolumab with platinum/fluoropyrimidine-based chemotherapy and the chemotherapy-free combination of nivolumab plus ipilimumab for patients with advanced, metastatic SCC with TPS ≥1 %.

In patients with locally advanced or metastatic SCC and EGC, the combination of pembrolizumab and chemotherapy (cisplatin + 5-FU) indicates significant benefit in OS with 12.4 vs. 9.8 months, HR 0.73 [95 % CI 0.62–0.86]; p<0.0001) independently from PD-L1 CPS and histology. Indeed, the subgroup of SCC and adenocarcinomas with CPS (combined positivity score) ≥10 benefited most from combination therapy (KEYNOTE 590) [5]. In September 2020, EU approval was granted for pembrolizumab in combination with platinum and 5-FU for SCC and EGC with CPS ≥10.

In patients with gastric, gastroesophageal junction, and esophageal adenocarcinoma and CPS ≥5, the combination therapy of nivolumab plus chemotherapy (FOLFOX/XELOX) resulted in significant improvements in OS (14.4 vs. 11.1 months, HR 0.71 [98.4 % CI 0.59–0.86]; p<0.0001) and progression-free survival (PFS) (7.7 vs. 6.0 months, HR 0.68 [98 % CI 0.56–0.81]; p<0.0001) vs. chemotherapy alone (CHECKMATE 649) [6]. Subsequently, the combination of nivolumab plus fluoropyrimidine/platin-based chemotherapy was approved for EGC patients with CPS ≥5 in Europe. Latest 36-month follow-up results presented at ASCO 2023 promote the OS benefit in the CPS ≥5 population: median OS 14.4 vs. 11.1 months, HR 0.70 [0.61–0.81] and median PFS 8.3 vs. 6.1 months HR 0.70, [0.60–0.81] [7]. Recent added approvals are chemotherapy with pembrolizumab in CPS ≥1 patients (KEYNOTE 859) and the addition of trastuzumab for personalized therapy in HER-2 positive/CPS ≥1 gastric and GEJ patients (KEYNOTE 811).

## Palliative second- and third-line immunotherapy

The multicenter phase III trial Attraction 03 evaluated the use of nivolumab in second-line therapy in SCC after progression on first-line therapy and demonstrates a significant survival benefit in overall survival for nivolumab monotherapy in comparison to chemotherapy (paclitaxel/docetaxel; median OS 10.9 vs. 8.4 months, HR 0.77 [95 % CI 0.62–0.96]; p=0.019) [8]. Based on these data, nivolumab was approved as a second-line treatment option for patients with advanced SCC regardless of PD-L1 expression status in Europe.

Except for the approval in MSI EGC patients, there is currently no approval for immunotherapy in patients with advanced EGC in second-line therapy. In the KEYNOTE 061 trial with pembrolizumab monotherapy vs. paclitaxel chemotherapy in patients with EGC and progression on first-line chemotherapy, the primary endpoint (superiority of OS) was not met. However, the data show a dependence of pembrolizumab efficacy on PD-L1-CPS score: patients with PD-L1-CPS ≥10 % expression demonstrate benefit of pembrolizumab therapy [9].

**Figure 1:**
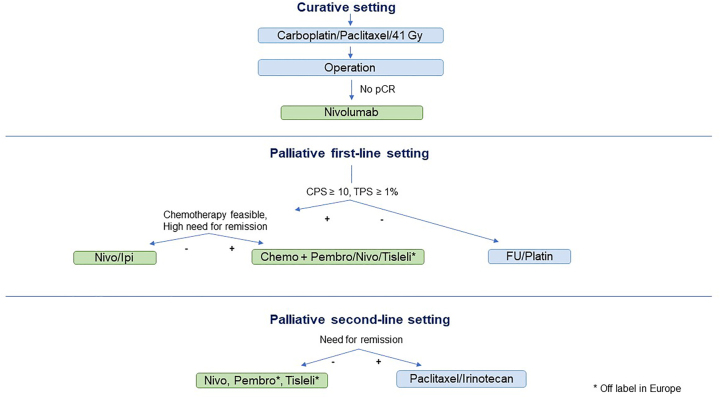
Potential therapy algorithm of SCC in Europe. PCR, Pathologic complete remission; CPS, Combined positive score; TPS, Tumor proportion score; Nivo, Nivolumab; Ipi, Ipilimumab; Pembro, Pembrolizumab; Nivo, Nivolumab; Tisleli, Tislelizumab.

**Figure 2:**
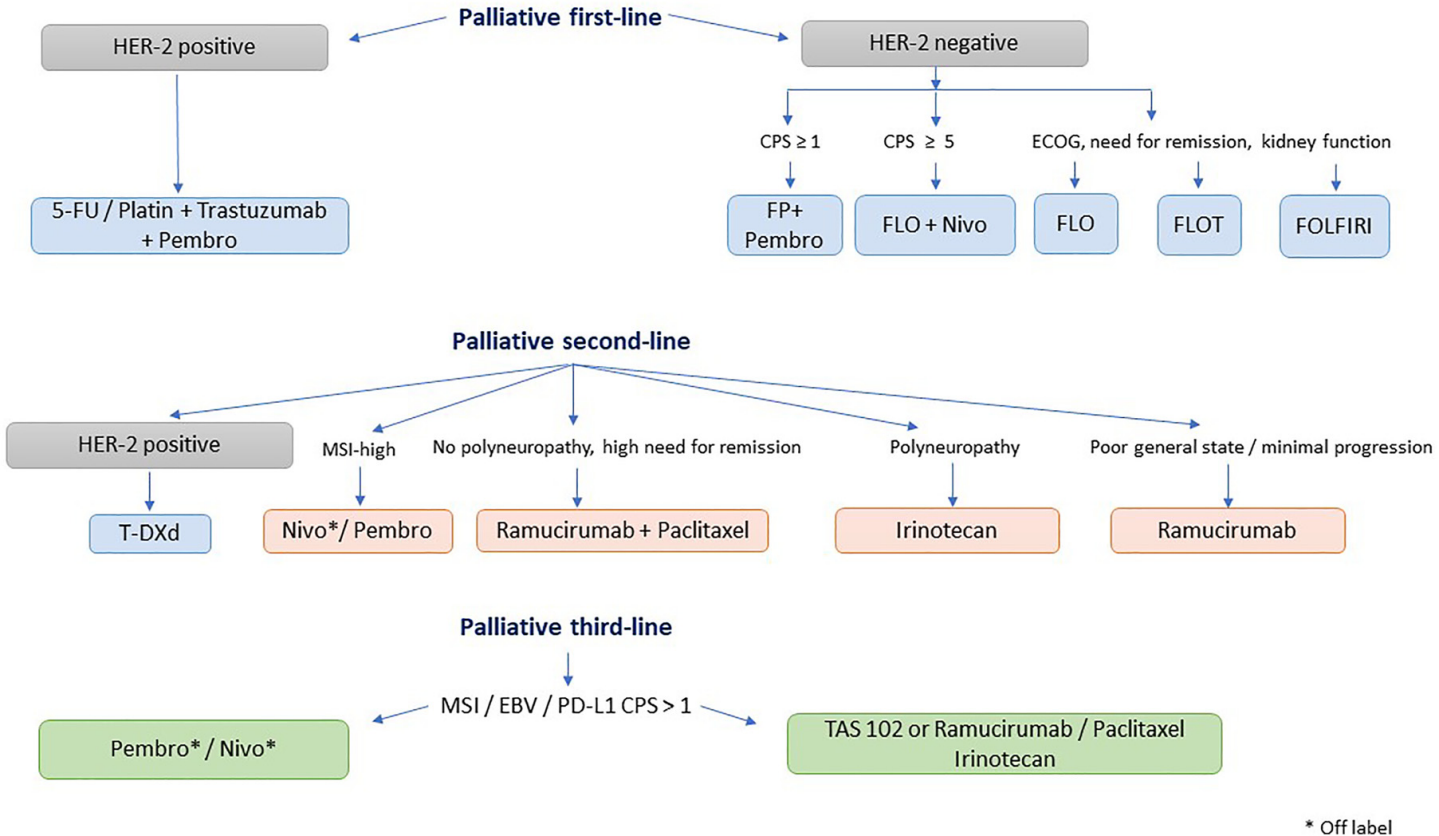
Potential therapy algorithm of adenocarcinoma of the esophagus in Europe [25]. Additional approval has been given for Pembro in CPS ≥1 patients (KEYNOTE 859) in HER-2 positive/CPS ≥1 gastric and GEJ patients (KEYNOTE 811). Pembro, Pembrolizumab; Nivo, Nivolumab; CPS, Combined positive score; MSI, Microsatellite instability; EBV, Epstein–Barr virus; GEJ-cancer, Esophagogastric junction cancer.

Potential therapy algorithms based on the current approvals for immune checkpoint inhibitors (ICI) therapy in Europe are presented in Figure 1 (SCC) and Figure 2 (EGC).

## Potential therapy options in the future

In the perioperative setting, the CROSS trial [10] and the FLOT regimen [11] represented the best perioperative multimodality concepts, which have been [2] and will further be improved by combinations with PD-1/PD-L1 inhibitors, currently under investigation: pembrolizumab in the perioperative treatment setting of EGC is being analyzed in the KEYNOTE 585 trial, where patients receive perioperative pembrolizumab plus chemotherapy (cisplatin + capecitabine/5-FU) vs. placebo plus chemotherapy [12]. A potential survival benefit of PD-L1 inhibitor durvalumab in the perioperative setting in patients with PD-L1-independent resectable EGC is investigated in a combination of durvalumab with FLOT vs. FLOT alone (MATTERHORN trial) [13]. Within the randomized phase II DANTE trial of the AIO (Arbeitsgemeinschaft Internistische Onkologie), patients with resectable localized EGC received the combination of atezolizumab (anti-PD-L1) plus chemotherapy (FLOT) compared with perioperative chemotherapy alone. First results show a feasible combination therapy and efficacy data are expected soon [14].

Various promising checkpoint inhibitors are under investigation in the palliative treatment of SCC and EGC: The combination of tislelizumab (PD-1 inhibitor) with chemotherapy (5-FU/platin-based) in the first-line therapy showed significant improvement of OS of 6.7 months in SCC patients with locally advanced/metastatic disease (median OS 17.3 vs. 10.6 months, HR 0.66 [95 % CI 0.54–0.80]; p<0.0001) (RATIONALE 306 trial) [15]. In the subgroup of patients with PD-L1-CPS ≥10 % expression, the OS benefit was particularly pronounced in the arm with checkpoint inhibition and chemotherapy vs. chemotherapy alone. Latest results of the interim analysis of the RATIONALE 305 trial at ASCO GI in January 2023 using tislelizumab placebo-controlled in first-line therapy in combination with chemotherapy (platin/5-FU) in patients with locally advanced or metastatic EGC demonstrated significant improvement in OS in the PD-L1 positive population (median OS 17.2 vs. 12.6 months, HR 0.74 [95 % CI 0.59–0.94], 1-sided p=0.0056). Combination of tislelizumab plus chemotherapy showed also longer PFS (median PFS 7.2 vs. 5.9 months, HR 0.67 [95 % CI 0.55–0.83]) [16]. Tislelizumab was also investigated in second-line therapy: after progression of SCC under prior chemotherapy with platinum and 5-FU, there was a significantly prolonged overall survival of tislelizumab monotherapy vs. chemotherapy (paclitaxel/docetaxel/irinotecan) (median OS of 8.6 vs. 6.3 months, HR 0.7 [95 % CI 0.57–0.85]; p=0.0001) (RATIONALE-302 trial). Again, the patient group with PD-L1-CPS ≥10 % expression showed greater benefit with a median OS of 10.3 vs. 6.8 months (HR 0.54 [95 % CI 0.36–0.79]; p=0.0006) [17].

As presented at ESMO 2021, further promising PD-1 inhibitors have potential to prolong OS in advanced SCC patients as investigated in Asian trials (Camrelizumab: ESCORT 1-trial, Sintilimab: ORIENT 15-trial, Toripalimab: JUPITER 06-trial) [18], [19], [20]. With respect to the Asian population, these results highlight the global efforts in implementing checkpoint inhibitors into standard first-line therapy regimens.

Another promising target for individualized therapy in advanced Her2-negative, Claudin-18.2-positive EGC (moderate-to-strong membrane staining in ≥75 % tumor cells) is the anti-Claudin-18.2 targeted antibody zolbetuximab (IMAB362), which specifically binds to the tight-junction component Claudin-18.2, which is expressed in malignant transformed gastric mucosa of diffuse and intestinal GC. At ASCO 2023, conclusive primary OS data of the combination of zolbetuximab with FOLFOX compared with placebo with chemotherapy were demonstrated (SPOTLIGHT trial). Median OS was improved by 2.7 months (median OS: 18.23 vs. 15.54 months, HR 0.750, p=0.0053), as well as PFS (median PFS: 10.61 vs. 8.67 months, HR 0.751, p=0.0066) [21].

With respect to Her2 (human epidermal growth factor receptor 2)-targeted therapy, combination of trastuzumab and chemotherapy was the best first-line standard of care for advanced/metastatic HER2-positive cancers patients [22]. With recent impressive results of the phase 3 KEYNOTE 811, trastuzumab and chemotherapy combined with pembrolizumab showed superior overall response rates (ORR 74.4 % (66.2–81.6) vs. placebo (51.9 % (43.0–60.7), 95 % CI 11.2–33.7, p=0.00006). The complete response rate (CR) was also beneficial in the triple combination (11.3 vs. 3.1 %), as well as the disease control rate (DCR, 95 % CI 96.2 % (91.4–98.8) vs. 89.3 (82.7–94.0) [23]. Based on the most recent presented survival benefits, pembrolizumab with chemo and trastuzumab was approved in patients with HER-2 positive/CPS ≥1 gastric and GEJ patients by the FDA and EMA. Various further approaches of Her2-targeted therapy are currently investigated addressing Her2-positive patients who do not benefit from trastuzumab and show progressive disease under prior trastuzumab therapy, including the antibody-drug conjugate trastuzumab-deruxtecan (T-Dxd), now approved in second line or later for HER2 + adenocarinoma. Combination of T-Dxd with immune checkpoint inhibitors show first promising results (DESTINY Gastric 03-trial) [24].

## Conclusions

Immunotherapy of SCC and EGC enriches the current therapy and is established as new standard treatment in almost every therapy line within a short period of time. Ongoing clinical trials further investigate different immune checkpoint inhibitors and address the remaining questions of individual responders/nonresponders to immunotherapy as well as the reuse of checkpoint inhibitors after progression to immunotherapy.
